# Incidence and risk factors of vertebral body collapse after posterior instrumented spinal fusion in elderly patients: An observational study

**DOI:** 10.1097/MD.0000000000031604

**Published:** 2022-11-04

**Authors:** Feng-Chen Kao, Yao-Chun Hsu, Tzu-Shan Chen, Yuan-Kun Tu, Pao-Hsin Liu

**Affiliations:** a Department of Orthopedics, E-Da Hospital, Kaohsiung, Taiwan; b School of Medicine for International Students, College of Medicine, I-Shou University, Kaohsiung, Taiwan; c Department of Orthopedics, E-Da Dachang Hospital, Kaohsiung, Taiwan; d Division of Gastroenterology, E-Da Hospital, Kaohsiung, Taiwan; e Department of Medical Research, E-Da Hospital, Kaohsiung, Taiwan; f Department of Medical Imaging and Radiological Sciences, College of Medicine, I-Shou University, Kaohsiung, Taiwan; g Department of Biomedical Engineering, College of Medicine, I-Shou University, Kaohsiung, Taiwan.

**Keywords:** compression fracture, osteoporoses, risk factor, spinal fracture, spinal fusions

## Abstract

This study investigates the incidence and risk factors of new vertebral body collapse (VC) after posterior instrumented spinal fusion in patients older than 70 years. This retrospective study analyzed the data of elderly patients who underwent posterior instrumented spinal fusion in the thoracolumbar spine between January 2013 and December 2017. The 2 subsamples comprised of patients who had experienced vertebral compression fracture (VCF) before the index spinal surgery (group 1, n = 324) and those who had not (group 2, n = 1040). We recorded and analyzed their baseline characteristics, their underlying comorbidities, and the details of their current instrumented spinal fusion. The incidences of new VC and screw loosening were recorded. In groups 1 and 2, the incidences of new VC were 31.8% and 22.7%, respectively, and those of new VC with screw loosening were 25.6% and 33%, respectively. The risk factor was upper screw level at the thoracolumbar junction (hazard ratio [HR] = 2.181, 95% confidence interval [CI]: 1.135–4.190) with previous VCF. The risk factors were age ≥ 80 years (HR = 1.782, 95% CI: 1.132–2.805), instrumented levels > 4 (HR = 1.774, 95% CI: 1.292–2.437), and peptic ulcer (HR = 20.219, 95% CI: 2.262–180.731) without previous VCF. Clinicians should closely monitor new VC after posterior instrumented spinal fusion in elderly patients with previous VCF with upper screw level at the thoracolumbar junction and in patients without previous VCF aged ≥ 80 years, with instrumented levels > 4 and peptic ulcer.

## 1. Introduction

Osteoporosis would increase susceptibility to fracture by deterioration of the bone architecture and low bone mineral density (BMD).^[1,2]^ The World Health Organization and Position Development Conference of the International Society for Clinical Densitometry define osteoporosis through a BMD T score of ≤−2.5 in postmenopausal women and men older than 50 years.^[3–5]^

Vertebral compression fractures (VCFs) are the most common type of fragility-related fracture.^[6]^ VCFs have multiple morbidities such as back pain, prolonged bed rest, decreased ambulation, and decreased pulmonary function.^[7,8]^ They can significantly lower a patient’s quality of life and increase the risk of mortality in elderly patients in whom adverse events occur.^[9,10]^

Degenerative and traumatic diseases of the osteoporotic spine become more likely as a person ages. Decompression and instrumented spinal fusion methods are used to treat these diseases in elderly patients.^[11]^ In treatments involving an aging spine, spine surgeons encounter discrepancies and challenges in spinal decompression and fusion surgeries.^[12]^

Spinal fusion methods include instrumented posterolateral lumbar fusion (PLF) and posterior lumbar interbody fusion (PLIF), as described by Cloward^[13]^ and have been widely employed for treating patients with degenerative lumbar diseases.^[14–16]^ Instrumented spinal fusion surgery using pedicle screws results in a much higher fusion rate than noninstrumented fusion alone and thus causes higher patient satisfaction^[14]^ and this technique is widely used for treating spinal degenerative diseases and other spinal disorders.^[17–19]^

**Figure 1.**
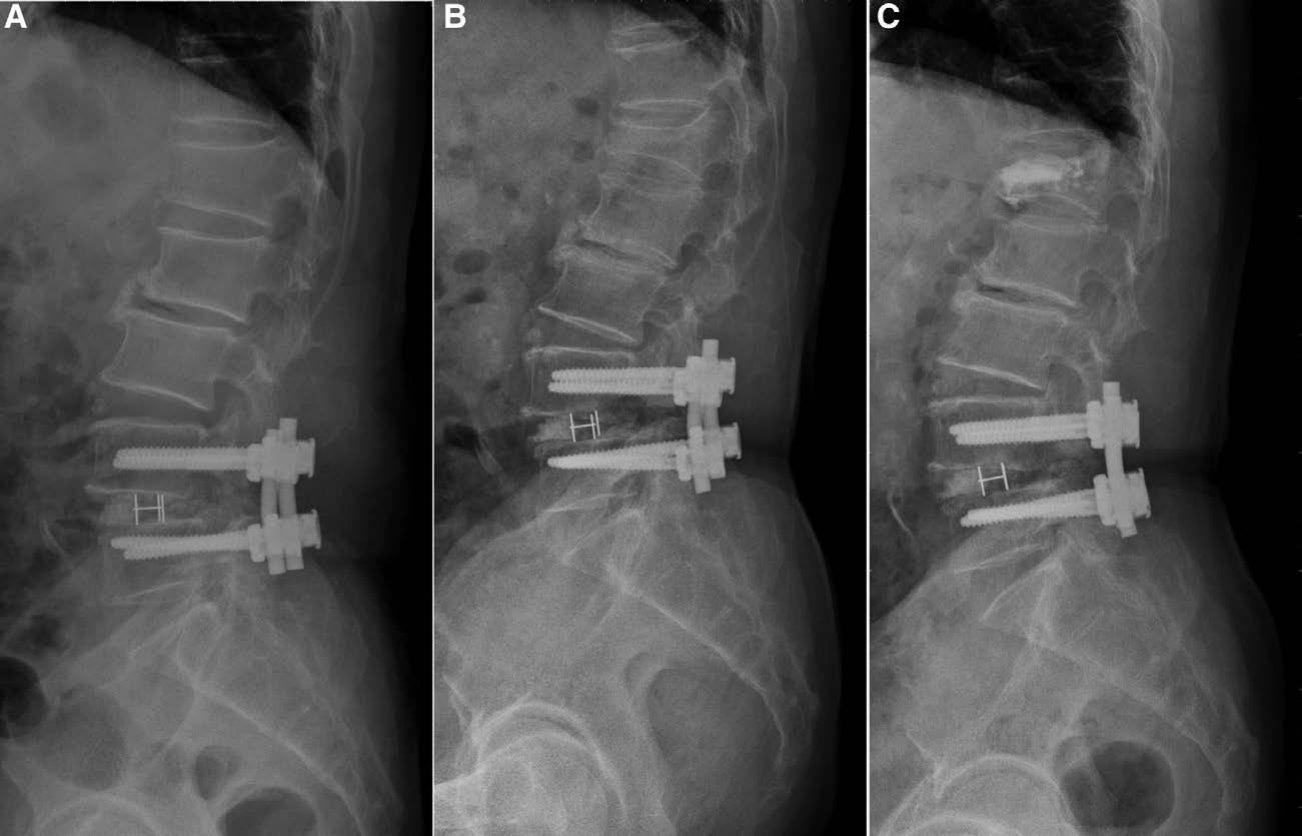
(A) L4–L5 instrumented spinal fusion was performed on a 73-year-old male patient. (B) New vertebral compression at the L1 level occurred during follow-up. (C) Vertebroplasty was performed for L1 compression fracture.

Pedicle screws will increase incidence of adjacent segment disease because of the corresponding immediate stiffness.^[16,20–22]^ The likelihood of pedicle-screw-related complications—such as loosening, migration, and back-out—increases when pedicle screws are applied in the osteoporotic spine.^[23]^ Vertebral body collapse (VC) such as new VCFs (Fig. 1) and fragility fracture at instrumented vertebral level are also more likely in patients who have undergone instrumented spinal fusion surgery.^[24]^

If the elderly patients received instrumented spinal fusion surgeries. Occurrence of vertebral body collapse will lead to unsatisfied surgical results and decreasing life quality even more mortality. However, the rates of these complications after spinal fusion surgeries in elderly patients remain unclear. Moreover, senile osteoporosis usually develops in men and women over age 70 years.^[25]^ Both trabecular and cortical bone are affected, leading to vertebrae fracture risk.^[26,27]^

Therefore, we conducted a retrospective study to determine the incidence of new VCs after instrumented spinal fusion in older adult patients (age ≥ 70 years old). We also investigated the risk factors of new VCs after posterior instrumented spinal fusion in patients older than 70 years.

## 2. Materials and methods

This retrospective study was conducted by reviewing patients’ medical records and radiography results from January 2013 to December 2017. We identified 1421 elderly patients who underwent instrumented PLF or PLIF for degenerative disease (including disk degeneration, recurrent disk herniation, spinal stenosis, spondylolisthesis, and degenerative scoliosis) in the thoracolumbar area. All patients were followed up until December 2018, with the mean follow-up period being 24.94 (12–52) months. The exclusion criteria were presence of malignancy, infective spondylitis, instrumented spinal fusion surgery for vertebral fractures, and erroneous data; 1364 patients were eligible for this study. We divided these 1364 patients into 2 groups based on whether they had a previous VCF before their instrumented spinal fusion surgery. Previous VCF was defined as the presence of a collapse or wedge deformity of the vertebral body in the thoracolumbar area as indicated in preoperative radiographic findings. Study groups 1 and 2 comprised 1040 patients without previous VCF and 324 patients with previous VCF, respectively.

We recorded the patients’ baseline characteristics—age, gender, body mass index, and baseline comorbidities—and their Charlson comorbidity index. We also identified whether the patients had undergone spinal fusion surgery previously. The patients of our study underwent surgery from January 2013 to December 2017. The spinal fusion levels when a pedicle screw was used were assessed by reviewing medical records and radiography results from before and after surgery. Radiography was performed before and after surgery (a routine protocol at postsurgery intervals of 1, 2, 3, 6, 9, and 12 months or at the time of reported intractable back or buttock pain). Instrumented levels were divided into ≥4 levels and <4 levels. Upper instrumented levels were divided into the thoracolumbar junction (T11, T12, and L1) and other-level groups.

**Figure 2.**
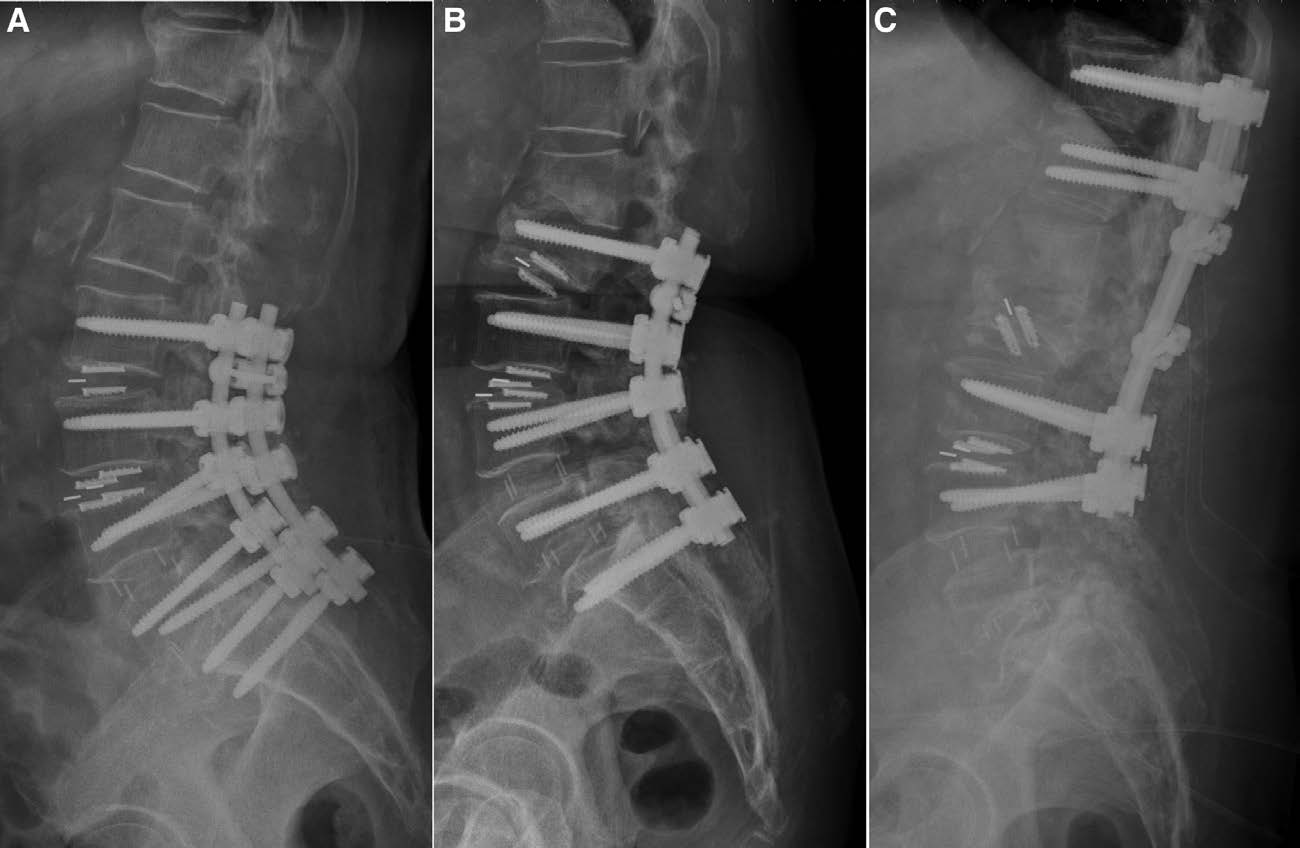
(A) L2–S1 instrumented spinal fusion was performed on a 77-year-old male patient. (B) Vertebral body collapse at the upper instrumented level and adjacent (one level above fusion segment) occurred during follow-up. (C) Revision spinal fusion surgery was performed with instrumentation extending to the T11 level.

**Figure 3.**
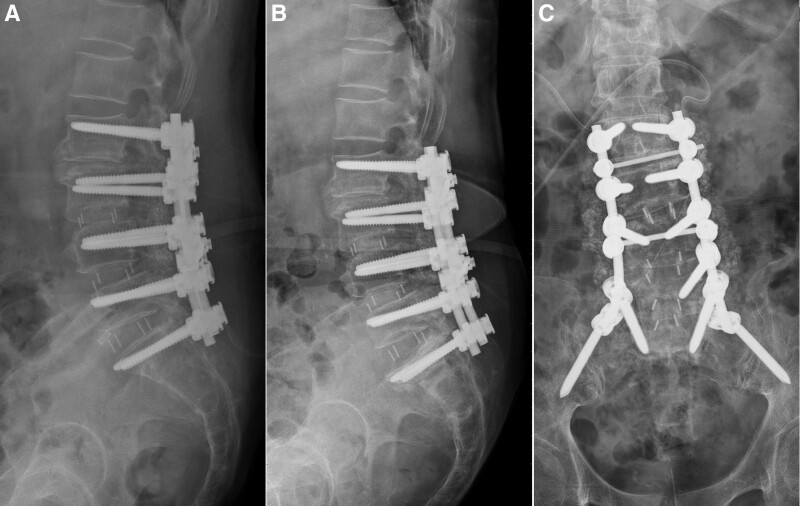
(A) L2–S1 instrumented spinal fusion was performed on a 73-year-old female patient. (B) Vertebral body collapse at the S1 level occurred during follow-up. (C) Revision spinal fusion surgery was performed with iliac screw instrumentation.

New VCs were identified by examining a patient’s thoracolumbar radiography or magnetic resonance imaging scans during the follow-up after instrumented spinal fusion surgery. We defined new VC as collapse of the vertebral body which including new VCF at noninstrumented vertebral level and VC at instrumented vertebral level. This collapse was detected by comparing pre- and postoperative thoracolumbar radiography results or on the basis of a signal change in the magnetic resonance imaging of the vertebral body; specifically, a weak signal on T1-weighted scans and a strong signal on T2-weighted scans indicated acute VC. New VC levels were defined according to their relationship with the fusion segments; for example, VCs could be one level above (or below) adjacent to the fusion segments, at the upper level (Fig. 2), or at the lowest level of the top instrumented level (Fig. 3). Data on treatment strategies, such as conservative treatments, vertebroplasty, kyphoplasty, and revision spinal fusion surgery, were obtained from medical records and radiographs; data on extended instrumented fusion levels were also obtained. The time interval between onset of new VCs and spinal fusion surgeries were also record. Besides, we record the number of cases received BMD exam and the number of cases received antiosteoporotic agents such as oral bisphosphonates, injected bisphosphonates (ibandronate or zoledronate), teriparatide, raloxifene, and denosumab during the period between 1 year before and 1 year after spinal fusion surgery.

**Figure 4.**
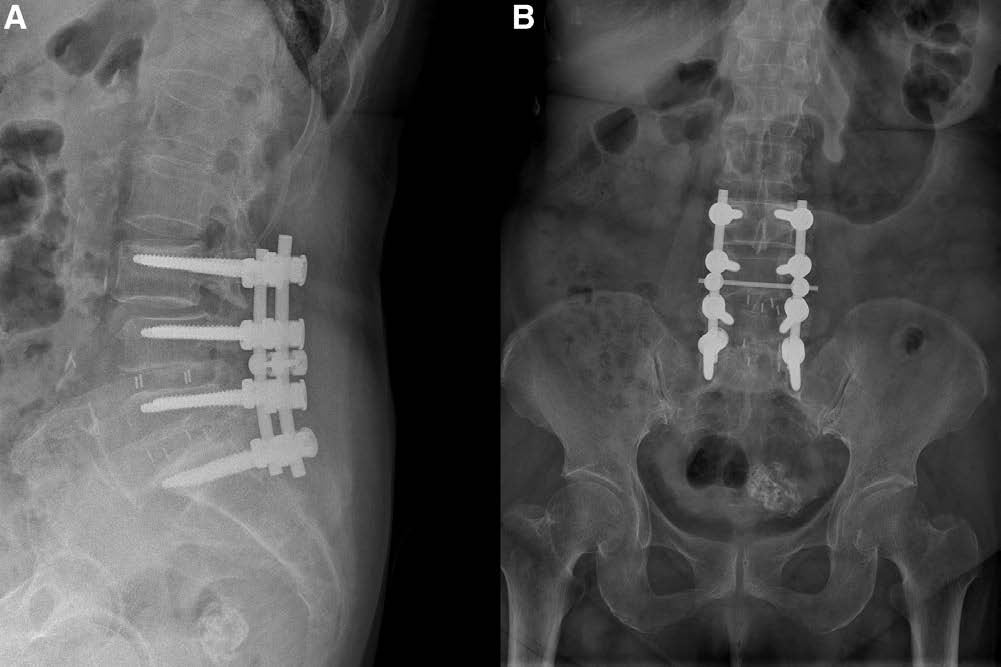
(A) L3–S1 instrumented spinal fusion was performed on a 73-year-old female patient. The anteroposterior view of the lumbar spine indicates a halo sign around the screws at the L3 and S1 levels. (B) Lateral view of lumbar spine indicated screw loosening at the upper (L3) and lowest (S1) instrumented levels of the fusion segment (halo sign around screws).

The transpedicle screws at the top and bottom levels of spinal fusion segments were evaluated with respect to the bone ingrowth condition of the vertebrae. Transpedicle screws were distinguished into 3 types, namely transpedicle screws, cement-augmented transpedicle screws (cement screws), and pedicle-screw-based dynamic stabilization systems (dynamic screws). Screw loosening was defined as the presence of a hollow shadow around the transpedicle screws (Fig. 4), which was evaluated by reading the series of radiographs obtained during follow-up.

Continuous and categorical variables are presented as the mean and the number and proportion, respectively. Risk factors for new VCs were identified using multiple logistic regression adjusted for age, gender, body mass index, previous spinal fusion surgery, instrumented level, upper instrumented level, and baseline comorbidity. Calculated results are expressed as ratios with 95% confidence intervals (CIs). A result was significant if *P* < .05. Analysis was performed using SAS (version 9.4; SAS Institute, Cary, NC). The acquisition and analysis of data used in this study were approved by the institutional re-view board of E-Da Hospital (no. EMRP-108-014). The IRB committee (name of the ethics committee: the institutional re-view board of E-Da Hospital) also approved that patient consents were not required in for this study. This study was performed in accordance with relevant guidelines and regulations of the IRB committee.

## 3. Results

### 3.1. Study group 1 without previous VCF

#### 3.1.1. Baseline characteristics of the study population.

Among the 1040 patients who underwent instrumented PLF or PLIF in the thoracolumbar area, 622 (59.8%) were women and 418 (40.2%) were men. Moreover, 189 (18%) patients had undergone instrumented PLF or PLIF before the index spinal fusion surgery. In total, 933 patients were aged between 70 and 80 years, and the mean age was 73.86 ± 2.59 years. In total, 107 patients were aged ≥ 80 years and the mean age was 82.60 ± 2.31 years.

The baseline characteristics and comorbidities of the 1040 patients are listed in Table 1. Three (0.29%) instances of mortality and 2 (0.19%) instances of infection were observed after index spinal fusion surgery.

**Table 1:** Base lines characteristics of patients without previous VCF (N = 1040).

	Fracture (N = 236)	No fracture (N = 804)	Total (N = 1040)
Age			
<80	74.00 ± 2.67 203 (86)	73.82 ± 2.57 730 (90.8)	73.86 ± 2.59 933 (89.7)
≥80	83.46 ± 2.59 33 (14)	82.26 ± 2.11 74 (9.2)	82.60 ± 2.31 107 (10.3)
BMI	26.16 ± 3.88	26.07 ± 3.84	26.09 ± 3.84
Height	154.85 ± 12.91	156.45 ± 7.84	156.08 ± 9.28
Weight	63.03 ± 11.30	63.91 ± 10.83	63.71 ± 10.94
Gender			
Female	153 (64.3)	469 (57.8)	622 (59.8)
Male	83 (34.9)	335 (41.3)	418 (40.2)
Anti-osteoporotic drug	50 (21)	76 (9.4)	126 (12)
Previous fusion	52 (22)	137 (17)	189 (18)
Upper screw level (T11,12 L1)	38 (16)	85 (10.5)	123 (11.7)
Cement screw	14 (5.9)	35 (4.3)	49 (4.7)
Number of instrumented fusion levels (≥4)	147 (62)	377 (46.8)	524 (50.2)
New VCF other than instrumented level	96 (40)	0	96 (9)
New VCF + screw loosening at instrumented levels	173 (73)	171 (21)	344 (33)
Upper screw new VCF	81 (34)	0	81 (7.7)
Loosening	86 (36.1)	74 (9.1)	160 (15.2)
Lower screw new VCF	19 (8)	0	19 (1.8)
Loosening	93 (39.1)	97 (11.9)	190 (18.1)
Vertebroplasty	47 (19.7)	2 (0.2)	49 (4.7)
Revision spinal fusion surgery			
Congestive heart failure	30 (12.6)	2 (0.2)	32 (3)
Peripheral vascular disease			
Cerebral vascular disease	4 (1.7)	4 (0.5)	8 (0.8)
Pulmonary disease	4 (1.7)	3 (0.4)	7 (0.7)
Peptic ulcer	5 (2.1)	1 (0.1)	6 (0.6)
Diabetes	9 (3.8)	30 (3.7)	39 (3.7)
Paraplegia	0	1 (0.1)	0
Renal disease	3 (1.3)	7 (0.9)	10
Metastatic cancer	0	1 (0.1)	1

BMI = body mass index, VCF = vertebral compression fracture

The BMD exams were performed in 283 (27.2%) cases. The antiosteoprotic agents were prescribed in 126 (12%) cases.

### 3.2. New VCs in the study cohort

In this study, 236 (22.7%) patients developed new VC after the index instrumented spinal fusion surgery. Of these patients, 81 (34%) had new VC at the upper instrumented vertebral level, and 19 (8%) had new VC at the lowest instrumented vertebral level. In total, we discovered 96 (40%) cases of adjacent new VC at levels other than the instrumented fusion segment.

The time interval between onset of new VCs and spinal fusion surgeries are 11.22 months (Table 1). There were 25.8% and 67.6% new VCs occurring after spinal fusion surgeries within 3 months and within 1 year respectively.

### 3.3. Risk factors for new VC after index instrumented spinal fusion surgery

In the unmatched data analysis, age ≥ 80 years (hazard ratio [HR] = 1.604, 95% CI: 1.034–2.487), upper screw level at the thoracolumbar junction (HR = 1.625, 95% CI: 1.075–2.457), instrumented level of ≥4 (HR = 1.859, 95% CI: 1.328–2.500), and peptic ulcer (HR = 17.403, 95% CI: 2.023–149.701) were significant risk factors for new VC after the index instrumented spinal fusion surgery (Table 3).

**Table 3:** Risk factors of new VCF analyzed by multiple variable logistic regression test (without previous VCF).

	Crude HR (95% CI)	*P* value	Adjusted HR (95% CI)	*P* value
Age group (<80)	ref			
Age group (>80)	1.604 (1.034–2.487)	.035	1.782 (1.132–2.805)	.013
BMI				
1 (<18.5)	1.80 (30.525–6.19)	.349	1.593 (0.453–5.609)	.468
2 (>24)	1.066 (0.766–1.485)	.704	1.013 (0)	.939
Upper screw level (T11 T12 L1 = 1)	1.625 (1.075–2.457)	.021	1.258 (0.810–1.954)	.307
Cement Screw	1.387 (0.734–2.624)	.314	1.279 (0.643–2.542)	.483
Dynamic screw	1.264 (0.780–2.047)	.341	1.254 (0.764–2.057)	.370
Instrumented levels *>* 4	1.859 (1.382–2.500)	<.001	1.774 (1.292–2.437)	<.001
Peripheral vascular disease	3.422 (0.213–54.92)	.385	2.483 (0.152–40.681)	.524
Cerebral vascular disease	3.453 (0.857–13.912)	.081	3.681 (0.885–15.317)	.073
Dementia	X	X	X	X
Peptic ulcer	17.403 (2.023–149.701)	.009	20.219 (2.262–180.731)	.007
Liver disease	X	X	X	X
Sex (female = 1)	1.317 (0.974–1.780)	.074	1.343 (0.976–1.849)	.071

CI = confidence interval, BMI = body mass index, HR = hazard ratio, VCF = vertebral compression fracture.

In the matched data analysis, age ≥ 80 years (HR = 1.782, 95% CI: 1.132–2.805), instrumented levels >4 (HR = 1.774, 95% CI: 1.292–2.437), and peptic ulcer (HR = 20.219, 95% CI: 2.262–180.731) were the corresponding risk factors (Table 3).

### 3.4. Screw-loosening rate at the upper and lowest instrumented spinal fusion levels

In total, 160 (15.2%) cases occurred at the upper instrumented vertebral level, and 190 (18.1%) cases occurred at the lowest instrumented vertebral level after the index spinal fusion surgery.

The combined rate of new VC and screw loosening after the index instrumented spinal fusion surgeries was 33% (344).

### 3.5. Study group 2 with previous VCF

#### 3.5.1. Baseline characteristics of the study population.

Of the 324 patients who had undergone instrumented PLF or PLIF in the thoracolumbar area, 244 (75.3%) were women, and 80 (24.7%) were men. Moreover, 72 (22%) patients underwent instrumented PLF or PLIF before the index spinal fusion surgery. In study group 2, 269 patients were aged between 70 and 80 years, and the mean age was 74.58 ± 2.61 years; 55 patients were aged ≥ 80 years, and the mean age was 82.34 ± 2.37 years.

The baseline characteristics and comorbidities of the 324 patients are listed in Table 2. We observed 3 (0.96%) instances of mortality and 1 (0.3%) instance of infection after the index spinal fusion surgery.

**Table 2:** Base lines characteristics of patients with previous VCF (N = 324).

	Fracture (N = 103)	No fracture (N = 221)	Total (N = 324)
Age			
<80	74.55 ± 2.65 84 (81.6)	74.61 ± 2.6 185 (83.7)	74.58 ± 2.61 269 (83)
≥80	81.83 ± 1.98 19 (18.4)	82.68 ± 2.57 36 (16.3)	82.34 ± 2.37 55 (17)
BMI	25.68 ± 4.39	25.56 ± 3.98	25.60 ± 4.11
Height	152.21 ± 16.76	152.8 ± 7.45	152.3 ± 11.25
Weight	59.38 ± 10.89	59.79 ± 10.98	59.81 ± 10.94
Gender			
Female	76 (73.8)	168 (76)	244 (75.3)
Male	27 (26.2)	53 (24)	80 (24.7)
Anti-osteoporotic drug	41 (39.8)	61 (27.6)	102 (31.5)
Previous fusion	28 (68)	44 (20)	72 (22)
Upper screw level (T11,12 L1)	28 (27.2)	28 (12.7)	56 (17.3)
Cement screw	8 (7.8)	22 (10)	30 (9.3)
Number of instrumented fusion levels (≥4)	63 (61.8)	107 (49.3)	170 (53.3)
New VCF other than instrumented level	54	0	0
New VCF + screw loosening at instrumented levels	83	47	130
Upper screw new VCF	41 (36.8)	0	41 (12.7)
Loosening	27 (26.2)	25 (11.3)	52 (16)
Lower screw new VCF	7 (6.8)	0	7 (2.2)
Loosening	34 (33)	22 (10)	56(17.3)
Vertebroplasty	34 (33)	1 (0.5)	35 (10.8)
Revision spinal fusion surgery	6 (5.8)	0	6 (1.9)
Congestive heart failure	2 (1.9)	3 (1.4)	5 (1.5)
Peripheral vascular disease	0	0	0
Cerebral vascular disease	0	3 (1.4)	3 (0.9)
Pulmonary disease	1 (1)	5 (2.3)	6 (1.9)
Peptic ulcer	1 (1	1 (0.5	2 (0.6)
Diabetes	5 (4.9)	8 (3.6)	13 (4)
Paraplegia	0	1	1 (0.3)
Renal disease	0	0	0
Metastatic cancer	0	0	0

BMI = body mass index, VCF = vertebral compression fracture.

The BMD exams were performed in 167 (51.5%) cases. The antiosteoprotic agents were prescribed in 102 (31.5%) cases.

### 3.6. New VC in the study cohort

In this study, 103 (31.8%) patients in study group 2 developed new VC after the index instrumented spinal fusion surgery. Of them, 41 (36.8%) had new VC at the upper instrumented vertebral level, and 7 (6.8%) had new VC at the lowest instrumented vertebral level. In total, 54 (16.7%) patients had adjacent new VC at levels other than the instrumented fusion segment.

The time interval between onset of new VCs and spinal fusion surgeries are 8.62 months (Table 2). There were 41.7% and 74.7% new VCs occurring after spinal fusion surgeries within 3 months and within 1 year respectively.

### 3.7. Risk factors for new VC after the index instrumented spinal fusion surgery

In the unmatched data analysis, upper screw level at the thoracolumbar junction (HR = 2.573, 95% CI: 1.430–4.632) and instrumented levels > 4 (HR = 1.611, 95% CI: 1.028–2.683) were significant risk factors for new VC after the index instrumented spinal fusion surgery (Table 4).

**Table 4:** Risk factors of new VCF analyzed by multiple variable logistic regression test (with previous VCF).

	Crude HR (95% CI)	*P* value	Adjusted HR (95% CI)	*P* value
Age group (<80)	ref			
Age group (>80)	1.162 (0.630–2.145)	.630	0.980 (0.502–1.914)	.954
BMI				
1 (<18.5)	2.741 (0.735–10.215)	.133	2.107 (0.533–8.326)	.288
2 (>24)	1.370 (0.811–2.316)	.239	1.362 (0.787–2.358)	.269
Upper screw level (T11 T12 L1 = 1)	2.573 (1.430–4.632)	.002	2.181 (1.135–4.190)	.019
Cement Screw	0.762 (0.327–1.774)	.528	0.776 (0.310–1.940)	.588
Dynamic screw	0.710 (0.141–3.577)	.678	0.803 (0.155–4.156)	.803
Instrumented levels *>*4	1.611 (1.028–2.683)	.038	1.316 (0.779–2.223)	.304
Peripheral vascular disease	X	X		
Cerebral vascular disease	X	X		
Dementia	X	X		
Peptic ulcer	2.157 (0.134–34.827)	.588	1.353 (0.074–24.743)	.838
Liver disease	X	X		
Sex (female = 1)	0.888 (0.519–1.519)	.888	0.973 (0.547–1.728)	.924

CI = confidence interval, BMI = body mass index, HR = hazard ratio, VCF = vertebral compression fracture.

In the matched data analysis, upper screw level at the thoracolumbar junction (HR = 2.181, 95% CI: 1.135–4.190) was the corresponding risk factor (Table 4).

### 3.8. Screw-loosening rate at the upper and lowest instrumented spinal fusion levels

Screw loosening occurred in 27 (26.2%) cases at the upper instrumented vertebral level and in 34 (33%) cases at the lowest instrumented vertebral level after the index spinal fusion surgery.

The combined rate of new VC and screw loosening after the index instrumented spinal fusion surgery was 25.6% (83).

## 4. Discussion

In the United States, 1.5 million fragility fractures occur annually, predominantly in postmenopausal women.^[28,29]^ In men older than 70 years, osteoporosis is referred to as age-related osteoporosis.^[30]^ Almost half of fragility fractures are VCFs. Once a VCF occurs, the possibility of a second new VCF within the next year is high.^[31]^ Moreover, spinal fusion surgery^[32]^ causes increased stress on the proximal segments from the rigid and a longer lever arm of fusion segments, leading to decreased BMD. Therefore, new VCs are more likely in patients who have received spinal fusion surgery, especially those who are elderly. In our stud, we found that VCs 25.8% and 41.7% within 3 months in cases with and without previous VCF, respectively. Within 1 year after spinal fusion surgeries, the incidence of new VCs increased to 67.6% and 74.7% in cases with and without previous VCF, respectively.

There were some reasons might lead to different analytic results between patients with and without previous VCF (Table 2). If patient got previous VCF, the period and severity of osteoporosis are more than patient without previous VCF. Besides, the saggital alignment might lead to unbalance condition after vertebral body collapse in patients with previous VCFs. We made 2 different cohort groups to evaluate the results in the 2 different background conditions.

DeWald reported an early complication rate of 13% (pedicle fractures and compression fractures) after multilevel spinal fusion for adult spinal deformity in patients older than 65 years.^[33]^ Li reported a vertebral fracture rate of 11% after lumbar instrumented circumferential fusion.^[34]^ Toyone reported a vertebral fracture rate of 24% in female patients and 2% in male patients following spinal fusion surgery for degenerative lumbar disease in patients older than 55 years.^[35]^ Our results confirm that new VCF is more likely to occur in women after instrumented spinal fusion surgery than in men. The reports mentioned only measured VCFs in noninstrumented vertebral bodies. We discovered that VCs may occur in instrumented vertebral bodies after spinal fusion surgery. Our study demonstrated that 103 (31.8%) and 236 (22.7%) of patients with and without previous VCF, respectively, developed new VC after the index instrumented spinal fusion surgery. The incidence of new VC was higher in the patients older than 80 years than patients younger than 80 years (HR = 1.782) without previous VCF before their instrumented spinal fusion surgery. Our study also demonstrated that instrumented spinal fusion level ≥ 4 was associated with a higher rate of new VCF (HR = 2.181 and 1.774) in both patients with and without previous VCF before their index spinal fusion surgery.

Factors contributing to proximal junctional failures have been reported including old age, male sex, osteopenia, thoracoplasty, preoperative comorbidities, rigid implant systems, preoperative hyperkyphotic thoracic alignment, preoperative kyphosis adjacent to the upper instrumented vertebra, high upper instrumented vertebral angle on lateral radiograph, instrumented vertebral level at L1 or L2, postoperative sagittal imbalance, sagittal imbalance associated with hip and knee degeneration, and acute corrections of sagittal malalignment.^[36,37]^ Our study also revealed that the instrumented vertebral level at the thoracolumbar junction (T11, T12, and L1) was associated with higher incidence of new VC (HR = 2.181) in patients with previous VCF before the index instrumented spinal fusion surgery. Beside, sacral fracture after lumbo-sacral fusion had be reported in literature,^[38,39]^ we found this fragility fracture type could happened after screws insertion to S1 body.

The mechanical force on the screw–bone interface has been reported to have adverse effects in patients with osteoporosis.^[40,41]^ Poor osseous quality also leads to increased risk of screw loosening in elderly patients.^[42]^ A high (52.73%) incidence of screw loosening^[43]^ at different levels of instrumentation has been reported (especially in patients older than 68.94 years). In our study, the screw-loosening rate was 33.4% and 33.6% after instrumented spinal fusion surgery in elderly patients with and without previous VCF, respectively. The clinical symptoms and long-term effects of screw loosening were not investigated in this study. We did not determine whether screw loosening progressed to cause further compression fractures. Long-term follow-up is required in future studies.

Complications can be treated through revision spinal surgeries, including decompression and proximal extension of the instrumentation^[36]^; vertebroplasty has been reported to be effective.^[44]^ Successful results in spinal fusion surgery have been reported when cement-augmented pedicle screws were applied to strengthen the fixation of the osteoporotic spine.^[12,45,46]^ However, our results indicate no association between cement-augmented pedicle screws and new VC after instrumented spinal fusion. We also discovered that VC can occur after the application of pedicle screws with cement stringing (Figure S1, Supplemental Digital Content, http://links.lww.com/MD/H846).

Our study demonstrated that peptic ulcer was associated with higher incidence of new VC (HR = 20.219) in patients without previous VCF before their instrumented spinal fusion surgery. The reason is that peptic ulcer increases the risk of osteoporosis.^[47,48]^ Identification and treatment of osteoporosis are essential for preventing VCs, especially in elderly patients. The incidence of new VC and screw loosening after instrumented spinal fusion surgeries was 25.6% and 33% in elderly patients with and without previous VCF, respectively. Our study showed that the BMD exams were performed in low percentage (27.2% and 51.5% of the elderly patients with and without previous VCF, respectively). Although these adverse events are related to osteoporosis, antiosteoporotic drugs were prescribed in only 31.5% and 12% of the elderly patients with and without previous VCF, respectively (Tables 1 and 2). Clinical physicians usually ignore the underling osteoporotic condition. Teriparatide treatment may an effective strategy for enhancing bone union and reducing screw loosening to manage lumbar degenerative disease in patients who are elderly or have osteoporosis.^[49,50]^ Underlying osteoporosis could be treated to reduce the risk of VC when performing instrumented spinal fusion surgery in older adult patients.

Our study has some limitations. First, this was a retrospective study. We could not determine the role of antiosteoporotic drugs in the prevention of VC after instrumented spinal fusion surgery. The BMD data, Ca levels and bone metabolism markers were lack due to those lab examinations usually not been required in spinal fusion surgery. We also did not determine the long-term effects of pedicle screw loosening on the clinical outcomes of spinal fusion surgery. However, the primary contribution of our study is our provision of an invaluable perspective for clinical physicians. Specifically, clinicians should pay attention to the potential adverse effects of osteoporosis in elderly patients who have undergone instrumented spinal fusion surgery.

More than 20% of elderly patients had developed new VC 52 months after posterior instrumented spinal fusion. For elderly patients without previous VCF, the risk of new VC was significantly higher in patients older than 80 years, who had peptic ulcer disease, and who had instrumented levels > 4. Among those elderly patients with previous VCF, the risk was higher when they had an instrumented level at the thoracolumbar junction.

## Acknowledgments

We thank Wallace Academic Editing for editing this manuscript.

## Author contributions

**Data collection:** Tzu-Shan Chen.

**Validation:** Yao-Chun Hsu.

**Writing – original draft:** Feng-Chen Kao.

**Writing – review & editing:** Yuan-Kun Tu, Pao-Hsin Liu.

## Supplementary Material


