# Supplementary material for: Incidence and risk factors of vertebral body collapse after posterior instrumented spinal fusion in elderly patients: An observational study
Source: Medicine (Baltimore). 2022 Nov 4;101(44):e31604. doi: 10.1097/MD.0000000000031604 (PMC9646654; doi:10.1097/MD.0000000000031604)

Supplemental Digital Content (Figure 1)

A. L1–L5 cement-augmented instrumented spinal fusion was performed on a 74-year-old female patient.

B. Vertebral compression at the lowest instrumented level (L5) of fusion segment occurred during follow-up.

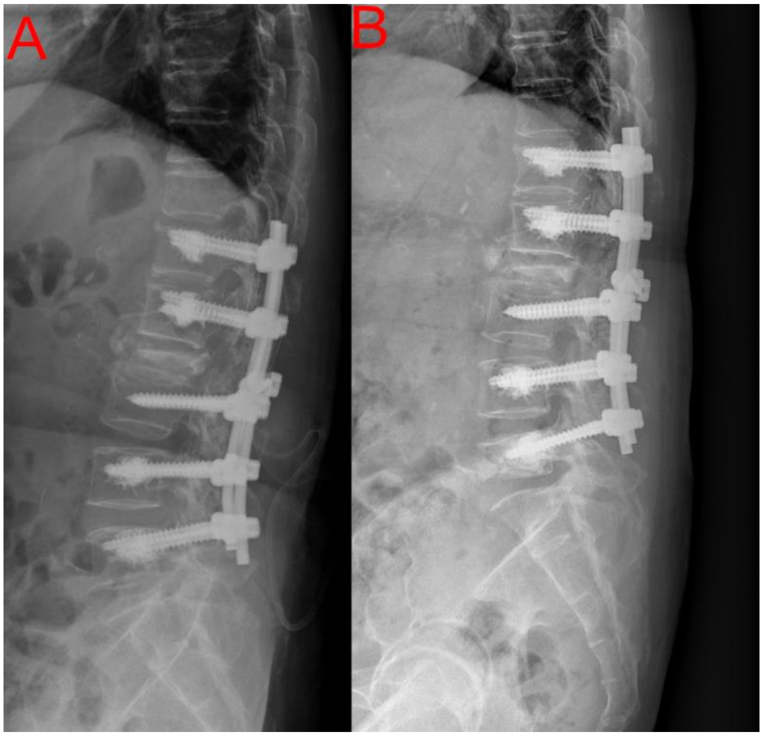

Supplement: Supplementary file 1 [file medi-101-e31604-s001.pdf]
